# Identification of HCN1 as a 14-3-3 client

**DOI:** 10.1371/journal.pone.0268335

**Published:** 2022-06-09

**Authors:** Colten Lankford, Jon Houtman, Sheila A. Baker

**Affiliations:** 1 Department of Biochemistry and Molecular Biology, University of Iowa, Iowa City, Iowa, United States of America; 2 Department of Microbiology and Immunology, University of Iowa, Iowa City, Iowa, United States of America; University of Milan, ITALY

## Abstract

Hyperpolarization activated cyclic nucleotide-gated channel 1 (HCN1) is expressed throughout the nervous system and is critical for regulating neuronal excitability, with mutations being associated with multiple forms of epilepsy. Adaptive modulation of HCN1 has been observed, as has pathogenic dysregulation. While the mechanisms underlying this modulation remain incompletely understood, regulation of HCN1 has been shown to include phosphorylation. A candidate phosphorylation-dependent regulator of HCN1 channels is 14-3-3. We used bioinformatics to identify three potential 14-3-3 binding sites in HCN1. We confirmed that 14-3-3 could pull down HCN1 from multiple tissue sources and used HEK293 cells to detail the interaction. Two sites in the intrinsically disordered C-terminus of HCN1 were necessary and sufficient for a phosphorylation-dependent interaction with 14-3-3. The same region of HCN1 containing the 14-3-3 binding peptides is required for phosphorylation-independent protein degradation. We propose a model in which phosphorylation of mouse S810 and S867 (human S789 and S846) recruits 14-3-3 to inhibit a yet unidentified factor signaling for protein degradation, thus increasing the half-life of HCN1.

## Introduction

Hyperpolarization activated currents (*I*_h_) carried by HCN1 channels play essential roles throughout the nervous system in tempering excitability, setting pacemaking activity, and coordinating signal integration [1–3]. Mutations that prevent ion conduction or alter gating are associated with high seizure susceptibility which can result in an extremely limited lifespan. Mutations affecting regions outside of the pore forming and voltage sensing domains may result in more mild generalized epilepsy or intellectual disability [4–6]. Given its role controlling excitability, it is unsurprising to find that HCN1 is implicated in many neurological functions. HCN1 plays a complex role in learning and memory with global knockout of HCN1 resulting in motor learning deficits while knockout of HCN1 selectively from the forebrain enhanced spatial reference memory [7, 8]. HCN1 knockdown in the CA1 region was shown to be anxiolytic [9]. In the forebrain HCN1 is inhibited by ketamine, contributing to the unique profile of this anesthetic and psychotropic drug [10, 11]. HCN1 is required in the olfactory bulb for activity-dependent neurogenesis [12]. And in the retina, HCN1 is required to shape the output of rod photoreceptors to prevent saturation of inner retinal circuitry [13–15]. HCN1 is an attractive pharmaceutical target [16]. The diversity of processes in which HCN1 participates makes it essential to uncover mechanisms of differential regulation. Further, the similarity between HCN1 and other HCN channels, including HCN4 the primary driver of cardiac rhythmicity [17, 18], highlights the need to identify possible drug targets specific for HCN1.

HCN1 is not a static channel but can be dynamically regulated. In hippocampal CA1 neurons, dendritic HCN1 participates in non-synaptic plasticity where HCN1 activity is downregulated to enhance spike probability and upregulated to reduce excitability [7, 19]. In addition, HCN1 channels are modulated in a homeostatic manner to prevent excessive signaling by strengthened synapses [20]. The neuromodulators neuropeptide Y and corticotrophin-releasing factor were shown to differentially modulate HCN1 activity in pyramidal neurons of the basolateral amygdala, downregulating and upregulating HCN1 respectively [21]. Dysregulation of HCN1 has also been observed and is associated with pathological states. Both acute and chronic HCN1 downregulation was observed in the hippocampus of a rat model of status epilepticus, leading to the speculation that dysregulation of HCN1 contributes to this condition [22]. Changes in HCN1 activity are also implicated in neuropathic pain. In layer 5 pyramidal neurons of the anterior cingulate cortex HCN1 was downregulated under chronic constriction injury [23]. Conversely, HCN1 has been shown to be upregulated in the periphery in models of neuropathic pain and has been shown to be a therapeutic target [24–28].

The activity, trafficking, and stability of HCN1 channels can be regulated by the accessory subunit, TRIP8b. TRIP8b binds to the cytoplasmic C-terminal tail in a bimodal manner, making contact with the membrane proximal Cyclic-Nucleotide Binding Domain (CNBD) and the terminal tripeptide Ser-Asn-Leu, thus bridging a 300 amino acid long intrinsically disordered region [29]. Differential regulation of HCN1 by TRIP8b can be accomplished through the expression of different TRIP8b splice isoforms [30–32]. Additionally, TRIP8b can be phosphorylated by CaMKIIa or PKA at a site involved in binding to the CNBD [33]. Two additional HCN1 binding proteins are the actin binding protein filamin A, and the ubiquitin ligase Nedd4-2, both of which can limit HCN1 surface expression [34, 35]. However, little is known about how the interaction between these proteins and HCN1 may be regulated. HCN1 itself is phosphorylated at multiple sites. Tyrosine phosphorylation or p38MAPK signaling can alter channel activity while PKC-dependent phosphorylation can alter surface expression [23, 36, 37]. Despite phosphorylation being implicated in HCN1 modulation, the effectors reading this post-translational modification are not known. 14-3-3 is a strong candidate for such an effector.

14-3-3 proteins are a conserved family of phosphoserine/phosphothreonine binding proteins ubiquitously expressed in eukaryotes and particularly enriched in the brain. There are over a hundred proteins that have been thoroughly validated as 14-3-3 client proteins. These include proteins participating in cellular functions as diverse as fatty acid metabolism and apoptosis [38, 39]. Bioinformatics predict the 14-3-3 ‘client-ome’ actually consists of several thousand proteins and is enriched in 2R-ohnologues [40]. HCN1 is an example of a 2R-ohnologue, which are small (two or four member) protein families arising from whole genome duplications. Among the 14-3-3 client proteins are multiple ion channels for which 14-3-3 may modulate channel assembly, trafficking, or gating [41–44]. One way that 14-3-3 can module channel trafficking is by masking an inhibitory di-arginine ER retention signal, as was shown to be the case for TASK-1 channels. HCN1 also contains a di-arginine ER retention signal although how that signal regulates HCN1 trafficking is currently unknown. These observations prompted us to investigate the possibility that 14-3-3 could be a phosphorylation-dependent regulator of HCN1.

Here we demonstrate that HCN1 is a novel 14-3-3 client protein and this interaction requires phosphorylation of two serines in the intrinsically disordered C-terminal tail of HCN1. Using cultured cells, we observed that the 14-3-3 interaction sites overlapped with a phosphorylation-independent site involved in degradation of HCN1.

## Materials and methods

The protocol for maintaining mice and obtaining tissue for experiments was approved by the University of Iowa IACUC.

### Molecular biology

eSpCas9(1.1)_No_FLAG_AAVS1_T2 was a gift from Yannick Doyon (Addgene plasmid # 79888; http://n2t.net/addgene:79888; RRID:Addgene_79888) [45]. The pSNAPf plasmid was obtained from New England Biolabs (cat. # N9183S) and the SNAP-tag was subcloned into a previously described plasmid encoding mouse HCN1 containing an HA-tag inserted into the extracellular loop between the S5 and S6 transmembrane domain (HA-HCN1) [46] to make SNAP-HCN1. pKanCMV-mRuby3-18aa-actin was a gift from Michael Lin (Addgene plasmid # 74255; http://n2t.net/addgene:74255; RRID:Addgene_74255) [47] and the mRuby3-tag was subcloned into the HA-HCN1 plasmid to make mRuby3-HCN1. While the HA-tag is not mentioned in this text, all HCN1 constructs used retain the HA-tag.

Homology directed repair templates for targeted knock in to the AAVS1 site were generated using AAVS1_Puro_PGK1_3xFLAG_Twin_Strep, a gift from Yannick Doyon (Addgene plasmid # 68375; http://n2t.net/addgene:68375; RRID:Addgene_68375) [45], as the base vector. SNAP-HCN1 was subcloned into this vector. In initial experiments SNAP-HCN1 expression was weak so we exchanged the PGK1 promotor for the CAG promotor from pCAG-mGFP, a gift from Connie Cepko (Addgene plasmid # 14757; http://n2t.net/addgene:14757; RRID:Addgene_14757) [48], to make AAVS1-CAG-SNAP-HCN1. The SNAP-tag was then exchanged for the mRuby3-tag from mRuby3-HCN1 to generate AAVS1-CAG-mRuby3-HCN1. 14-3-3 site mutations were made individually using site-directed mutagenesis and the double or triple mutant was then stitched together using in-fusion cloning to generate mRuby3-HCN1_Δ3, 14-3-3_ and SNAP-HCN1_Δ804–814; Δ861–871_. The cloning of serine to alanine substitutions into AAVS1-CAG-SNAP-HCN1 was performed by Genscript Cloning Services.

pDisplay-AP-CFP-TM was a gift from Alice Ting (Addgene plasmid # 20861; http://n2t.net/addgene:20861; RRID:Addgene_20861) [49]. The C-terminus of HCN1 was subcloned behind the transmembrane domain then the extracellular CFP tag was swapped for the SNAP-tag making the SNAP-TM-HCN1_CT_ (CT_598-910_) reporter from which truncations were made up to the transmembrane domain (control reporter).

All sequences were validated by Sanger sequencing (Iowa Institute of Human Genetics, University of Iowa, Iowa City, IA or Eurofins Genomics, Louisville, KY). Novel plasmids are available upon request–AAVS1-CAG-mRuby3-HCN1 (wild-type and mutants), AAVS1-CAG-SANP-HCN1 (wild-type and mutants), and SNAP-TM-HCN1 (various truncations and targeted mutations).

### Cell culture

HEK293 (ATCC CRL-3216) were cultured in DMEM (Gibco cat. # 11965092) supplemented with 10% fetal bovine serum (Gibco cat. # 26140079), 100 U/mL penicillin, 100 μg/mL streptomycin (Gibco cat. # 15140122), and 0.5 μg/mL amphotericin B (Gibco cat. # 15290018). Cells were maintained at 37°C with 5% CO_2_ in a humidifying incubator. Cells were passaged every two to three days and all experiments were performed prior to passage 25. Knock in cell lines were generated by co-transfecting cells 80% confluent in 6-well plates with 2 μg of AAVS1 homology directed repair template plasmid (wild-type and mutant versions of AAVS1-CAG-SNAP-HCN1 and AAVS1-CAG-mRuby3-HCN1) and 0.5 μg of AAVS1 targeted Cas9 plasmid (eSpCas9(1.1)_No_FLAG_AAVS1_T2). 24 hours after transfection, cells were treated with a high dose of puromycin, 5 μg/mL. After 48 hours, cells were switched to a maintenance dose of 2 μg/mL Puromycin which was maintained throughout. For transient transfections, 8 μg plasmid/10 cm plate or 2 μg plasmid/well of 6-well plate was used and cells harvested 24 hours post-transfection. All transfections were performed using Lipofectamine 2000 (ThermoFischer Scientific cat. # 11668027) at a 2:1 Lipofectamine (μL) to plasmid DNA (μg) ratio as described by the manufacturer.

### 14-3-3ζ purification

pPROEX-HTb vector for expression of human _6His_14-3-3ζ was a gift from Dr. Christian Ottmann [50]. This plasmid was transformed into BL21(DE3) *E*. *coli* and protein expression was induced with 1 mM isopropyl β-D-1-thiogalactopyranoside (IPTG) at mid-log phase. Induced cells were lysed using B-PER Bacterial Protein Extraction Reagent (ThermoFischer Scientific cat. # 90084) and purified using HisPur Ni-NTA resin (ThermoFisher Scientific cat. # 88221) or a HisTrap HP column (GE Healthcare). Purified _6His_14-3-3ζ was then dialyzed into PBS. Quality of the purification was assessed by SDS-PAGE and Coomassie staining, and protein concentration was measured by absorbance at 280nm.

### Electrophysiology

Current recordings from HEK293 cell lines expressing mRuby3-HCN1 or mRuby3-HCN1_Δ3, 14-3-3_ were performed by Eurofins Discovery (St Charles, MO, USA). Recordings were made using the Qpatch automated patch clamp system. Recording conditions: Intracellular Solution (mM): 70 KF, 60 KCl, 15 NaCl, 5 HEPES, 5 EGTA, 4 MgATP, 0.01 cAMP (pH 7.3 by KOH). Extracellular Solution (mM): 110 NaCl, 30 KCl, 1 MgCl_2_, 1.8 CaCl_2_, 10 HEPES, 5 Glucose (pH 7.35 by NaOH). Current recordings at tests pulses from –30 to –130 mV were provided, as was the capacitance of each cell. From the data provided, the current density and percentage of maximal current were obtained and plotted against the voltage. Plots were fit to a Boltzman sigmoidal. All electrophysiology data are presented as mean ± standard error. For comparisons between cell lines, the maximal current density was compared using t-test.

### Antibodies and western blotting

All antibodies used are detailed in Table 1 and western blotting was performed using a standard protocol. Premade 4–20% TGX gels with either the 10-well 50 μL or 15-well 15 μL formats (Bio-Rad cat. # 4561094 and 4561096) were used in all instances. Sample buffer was prepared using 4X LDS buffer (ThermoFischer Scientific cat. # NP0007) and 10X reducing agent (ThermoFischer Scientific cat. # NP0009). SDS-PAGE was performed with a tris-glycine running buffer (25 mM Tris, pH 8.3, 192 mM glycine, 0.1% SDS). Transfer was then performed with a tris-glycine transfer buffer (25 mM Tris, pH 8.3, 192 mM glycine, 15% methanol, 0.01% SDS). Proteins were transferred to low fluorescence PVDF membrane (Millpore Sigma cat. # IPFL00010). REVERT total protein stain was used to detect and quantify transferred proteins. Prior to immunolabeling, membranes were blocked with Intercept blocking buffer (LI-COR cat. # 927–60001). Primary antibody was diluted in a 50/50 mixture of blocker and wash buffer (39 mM Tris, pH 7.5, 300mM NaCl, 0.1% Tween-20) and incubated on membrane overnight at 4°C. Membranes were washed with wash buffer prior to and after 1 hour secondary antibody incubation also diluted in a 50/50 mixture of blocker and wash buffer. For co-immunoprecipitation experiments blots were imaged using the ImageQuant LAS 4000 (GE Healthcare). Image adjustments to brightness and contrast were made using Adobe PhotoShop 2020. All other blots were imaged using a LI-COR Odyssey Fc imager and images were quantified and edited using the Image studio software (Ver 5).

**Table 1:** Antibodies.

Antibody	Source	Application	Figure
rabbit anti-HCN1	Pan et al., 2014 [51]	western blotting against HCN1 in co-IP and pulldown experiments (1:3000)	2
mouse anti-HCN1	UC Davis/NIH NeuroMab Facility Cat# N70/28, RRID:AB_2877279	western blotting against mRuby3-HCN1 (1:1000)	3
rabbit anti-14-3-3	Santa Cruz Biotechnology Cat# sc-629, RRID:AB_2273154	western blotting against 14-3-3 in co-IP experiments (1:1000)	2A
mouse anti-14-3-3	Thermo Fisher Scientific Cat# MA5-12242, RRID:AB_10986852	Immunoprecipitating antibody for co-immunoprecipitation experiments (2.5ug/200uL of lysate)	2A
mouse anti-GFP	Takara Bio Cat# 632380, RRID:AB_10013427	Control antibody for co-immunoprecipitation experiments (2.5ug/200uL of lysate)	2A
rabbit anti-SNAP	New England Biolabs Cat# P9310S, RRID:AB_10631145	western blotting against SNAP-TM reporters and SNAP-HCN1 (1:1000)	4, 5
mouse anti-NKA	Santa Cruz Biotechnology Cat# sc-58628, RRID:AB_781525	western blotting in surface biotinylation experiments (1:1000)	3D, 5A
mouse anti-GAPDH	DSHB Cat# DSHB-hGAPDH-2G7, RRID:AB_2617426	western blotting in surface biotinylation experiments (1:100)	3D, 5A
TruBlot anti-mouse-HRP	Rockland Cat# 18-8817-30, RRID:AB_2610849	secondary antibody used in co-IP experiments (1:1000)	2A
goat anti-mouse800	LI-COR Biosciences Cat# 925–68071, RRID:AB_2721181	secondary antibody (1:20,000)	
goat anti-rabbit680	LI-COR Biosciences Cat# 925–32210, RRID:AB_2687825	secondary antibody (1:20,000)	
goat anti-mouse800	Thermo Fisher Scientific Cat# SA5-35521, RRID:AB_2556774	secondary antibody (1:20,000)	
goat ant-rabbit 680	Thermo Fisher Scientific Cat# 35568, RRID:AB_614946	secondary antibody (1:20,000)	

### Co-immunoprecipitation and pulldown assays

All samples were homogenized on ice in lysis buffer (1% Triton X-100 in PBS: 137mM NaCl, 2.7 mM KCl, 10 mM Na_2_PO_4_, 1.8 mM KH_2_PO_4_, pH 7.4) supplemented with cOmplete Mini, EDTA free protease inhibitor cocktail and PhosSTOP phosphatase inhibitor cocktail (Millipore Sigma cat. # 11836170001 and 4906845001). For tissue preparation, wildtype (C57Bl6/J) or HCN1 knockout mice as described in [51] were used. Whole brain was removed and homogenized in 5 mL of lysis buffer and 4–6 retina were homogenized in 1 mL of lysis buffer. HEK 293 cells were harvested by addition of lysis buffer directly to the culture plate. For full-length HCN1, transfected HEK293 cells on a 10 cm plate were lysed with 1 mL of lysis buffer. For the SNAP-TM reporters, transfected HEK293 cells on 6-well plates were lysed with 400 μL of lysis buffer per well. BCA assays (ThermoFischer Scientific cat. # 23227) were used to measure protein concentration.

For the 14-3-3 immunoprecipitation, 2.5 μg of anti-14-3-3 or anti-GFP antibodies were immobilized on 20 μL of protein A/G for two hours at 4°C. 200 μL of lysate (300–400μg total protein) was added to antibody-bound resin overnight at 4°C and washed with lysis buffer. Protein was eluted with 2X reducing sample buffer.

For 14-3-3 pulldowns, _6His_14-3-3ζ was incubated with Ni-NTA resin in 5% BSA dissolved in lysis buffer supplemented with 25 mM imidazole for 3 hours at 4°C; 5 mg of _6His_14-3-3ζ was used for pulldown from brain, 0.5 mg for pulldown from retina and HEK293 cells expressing full-length HCN1, 0.1 mg for pulldown of SNAP-TM-HCN1_CT_ reporters. For the control, Ni-NTA resin was treated identically without the addition of _6His_14-3-3ζ. 1.5 mL of whole brain lysate (30% of total brain lysate), 400 μL of retinal lysate (40% of total lysate from 6 retina– 300–400 μg), 400 μL of HCN1 expressing HEK cell lysate (50% of lysate from 10 cm dish– 300–500 μg), or 300 μL of reporter expressing HEK293 cell lysate (95% of lysate from well of 6-well plate– 300–400 μg) was added to control or _6His_14-3-3ζ conjugated resin and incubated overnight at 4°C. Resin was then washed with lysis buffer supplemented with 25 mM imidazole and protein was eluted with 250 mM imidazole in 8% SDS in 0.1 M Tris pH 7.8.

For pulldowns using the SNAP-TM reporters, the amount of each reporter pulled down was compared using a t-test or 1-way ANOVA followed by Dunnett’s multiple comparison to compare the truncated or mutant reporters to control (CT_598-910_ or CT_731-910_). All pulldown data presented as mean ± standard deviation.

### Surface biotinylation

HEK293 cells were briefly washed with cold PBS prior to incubation with 2 mg/mL Sulfo-NHS-SS-Biotin (APExBIO cat. # A8005) in cold PBS for thirty minutes on ice. The reaction was quenched by the addition 50 mM Tris-HCl, 150 mM NaCl, pH 7.5). Cells were briefly washed with PBS prior to addition of lysis buffer. For each replicate, equal amounts of protein (200–300 μg) for each experimental sample was added to 75 μL of High capacity Neutravidin agarose (ThermoFischer Scientific cat. # 29202) and incubated at 4°C overnight. Resin was washed with lysis buffer supplemented with 0.5% SDS. Protein was eluted by incubation for 5 minutes at 75°C in 2X reducing sample buffer prepared from 4X LDS sample buffer and 10X reducing agent. Eluted protein was then examined by western blot. T-test was used to compare mRuby3-HCN1 and mRuby3-HCN1_Δ3, 14-3-3_. 1-way ANOVA followed by Dunnett’s multiple comparison was used to compare SNAP-HCN1_Δ804–814; Δ861–871_ and SNAP-HCN1_S810A; S867A_ to SNAP-HCN1. All data presented as mean ± standard deviation.

### Pulse-chase experiments

Stable HEK293 cell lines were grown to confluency on a 35mm dish. The day before the pulse-chase experiment, cells were trypsinzied, resuspended in 10 mL of media, and 0.5 mL of the cell suspension was plated in each well of a 24-well plate. The following day cells were treated with 1 μM SNAP-cell TMR-Star (New England BioLabs cat. # S9105S) in complete culture media for 45 minutes at 37°C; for the chase, media was replaced with media containing 3 μM SNAP-Cell block (New England BioLabs cat. # S9106S) and cells maintained at 37°C. Aliquots of cells were harvested immediately at the end of the pulse or at 4, 8, 12, and 24 hours into the chase. Lysates were immediately frozen and processed once the final timepoint was collected. Lysates were quantified by BCA assay and equal total protein concentrations were analyzed by western blotting against the SNAP-tag with membranes imaged for SNAP-HCN1 conjugated SNAP-Cell TMR-star (600 nm channel on LI-COR Odyssey FC) prior to blocking.

The labeled SNAP-HCN1 fluorescent signal was normalized to SNAP-HCN1 western blot intensity. This value was then normalized to the 0-hour chase timepoint to give the % labeled SNAP-HCN1. The half-life was calculated by fitting the data to a one-phase decay curve. 2-way ANOVA followed by Dunnett’s multiple comparison was used to compare SNAP-HCN1_Δ804–814; Δ861–871_ and SNAP-HCN1_S810A; S867A_ to SNAP-HCN1. Data presented as mean ± standard deviation.

## Results

**Fig 1:**
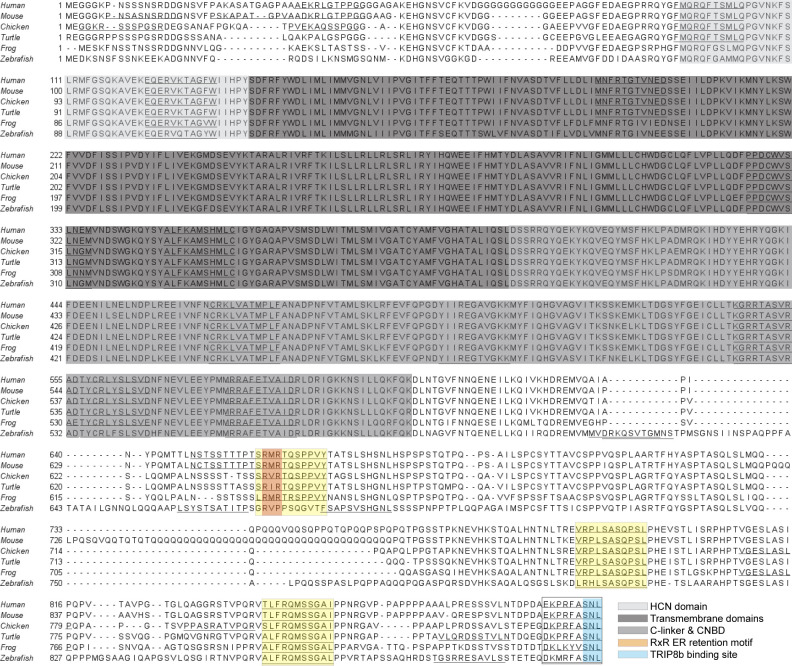
The C-terminus of HCN1 contains multiple predicted 14-3-3 binding sites. Sequence alignment of HCN1 from five vertebrate species. The structured domains of HCN1 are shaded as follows: light gray, HCN domain; dark gray, transmembrane domains; and medium gray, gating ring (C-linker and Cyclic Nucleotide Binding Domain (CNBD)). The RxR ER retention motif is colored in orange and the TRIP8b binding site in cyan. 14-3-3Pred predicted 14-3-3 binding sites are underlined and the three sites that were tested in this study are further shaded in yellow.

We analyzed the sequence of HCN1 using 14-3-3Pred, a prediction tool for the identification of phospho-peptides likely to serve as 14-3-3 ligands [52]. Several potential 14-3-3 binding sites (underlined, Fig 1) were predicted. 14-3-3 binding sites need to be accessible to kinases and 14-3-3 proteins so are often found in disordered domains of client proteins. To focus our efforts, we selected just the predicted sites within HCN1 that occur in disordered regions of HCN1 and are conserved among different vertebrates, thus leaving four sites for further evaluation. One of those four peptides overlaps the terminal Ser-Asn-Leu tripeptide required for binding to the accessory subunit TRIP8b thus would be inaccessible to 14-3-3 *in vivo* [29]. The three remaining sites (shaded in yellow, Fig 1) which we refer to as peptides A, B, and C (A = ^653^SRMRTQ**S**PPVY, B = ^804^VRPLSA**S**QPSL, C = ^861^TLFRQM**S**SGAI, the serine marked in bold is the predicted phosphorylated residue), are in the long, intrinsically disordered portion of the HCN1 C-terminal tail which contains 37 serines and 30 threonines (49% of all serines and threonines in HCN1). Intriguingly, one of the predicted peptides (peptide A) overlaps the ER retention motif in HCN1.

### 14-3-3 interacts with HCN1

**Fig 2:**
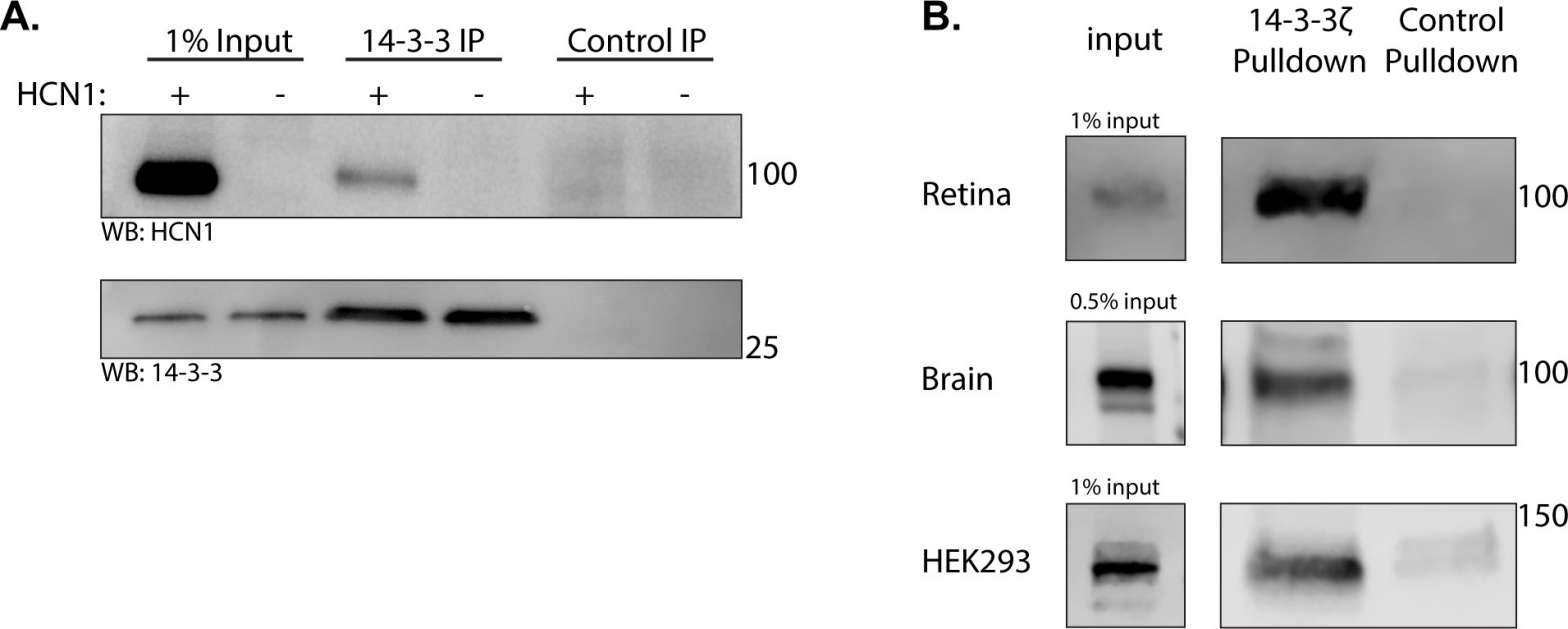
14-3-3 interacts with HCN1. **A)** Western blotting for HCN1 and 14-3-3 following 14-3-3 IP or control GFP IP from wild-type and HCN1 KO retina. **B)** Western blotting for HCN1 following pulldown with recombinant 14-3-3ζ or resin only control from mouse retina, whole brain, and HCN1 expressing HEK293 cells.

To determine if HCN1 and 14-3-3 interact, we used a pan-14-3-3 antibody to immunoprecipitate 14-3-3 from retina lysates. HCN1 was co-immunoprecipitated from wild-type retina but not HCN1 knockout retina which was used to ensure specificity of the HCN1 signal. An anti-GFP antibody was used as a negative control to ensure HCN1 was not binding non-specifically to the beads or mouse IgG (Fig 2A). Reciprocal co-immunoprecipitations using anti-HCN1 antibodies did not pulldown 14-3-3. The epitopes for both the commercially available anti-HCN1 (clone 70/28) and an in-house anti-HCN1 antibody [51] are in the C-terminus of HCN1 so the anti-HCN1 antibodies may not have access to 14-3-3 bound HCN1. Next, we performed pull-down assays using recombinant _6His_14-3-3ζ (hereafter referred to simply as 14-3-3). 14-3-3 immobilized on Ni-NTA beads, but not the unconjugated control beads, pulled down HCN1 from whole brain lysate, retinal lysate, and lysate from HCN1 expressing HEK293 cells (Fig 2B). We conclude that HCN1 can interact with 14-3-3 although it is not possible from these assays to determine if this interaction is direct.

### 14-3-3 interaction sites are not required for HCN1 to traffic to the cell surface

**Fig 3:**
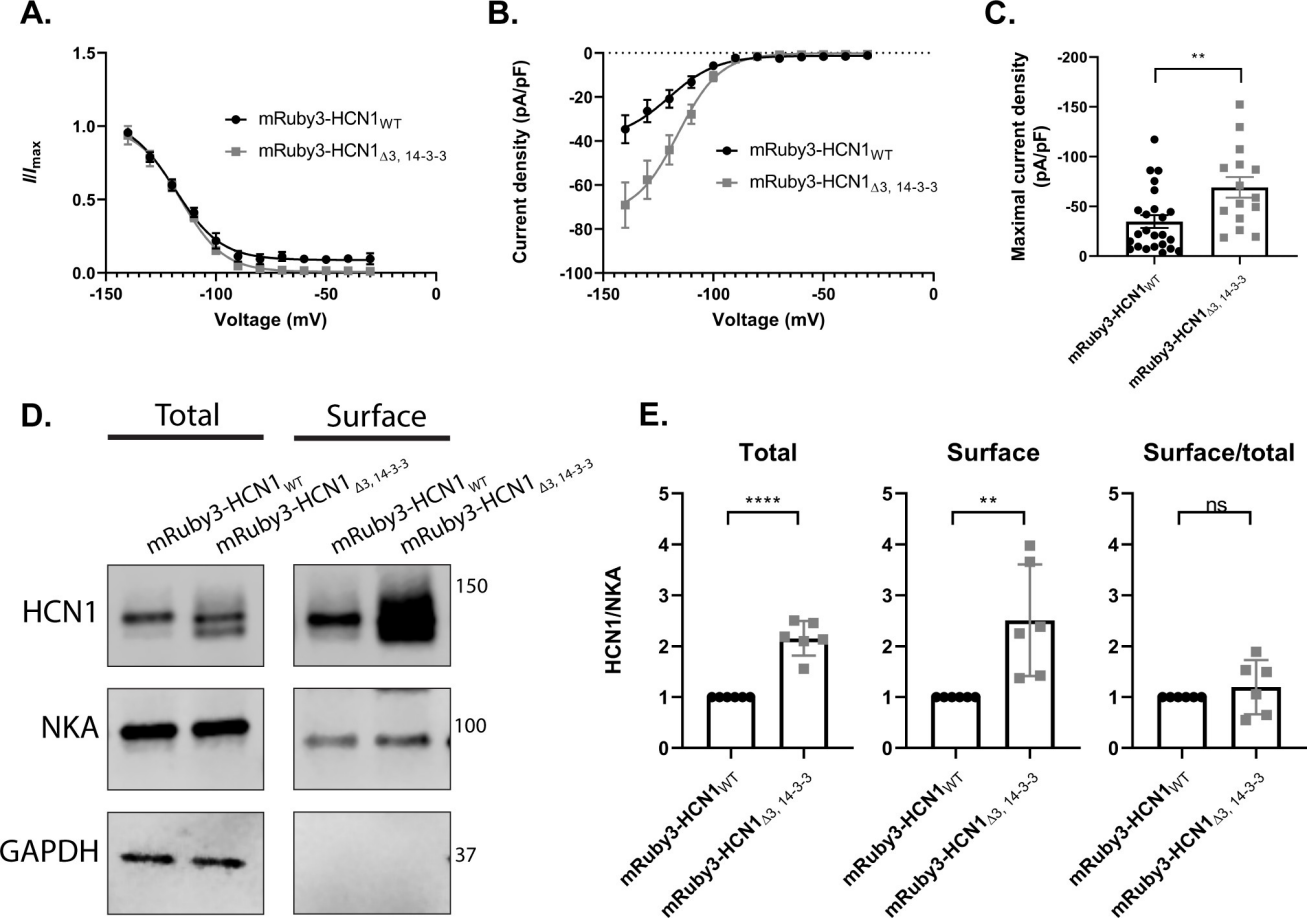
Mutating 14-3-3 interaction sites enhances HCN1 protein level in stable HEK293 cell lines. Activation voltage **(A)**, current density **(B)**, and maximal current density of *I*_h_ **(C)**, recorded from HEK293 cell lines expressing mRuby3-HCN1 (black) or mRuby3-HCN1_Δ3, 14-3-3_ (grey). **D)** Representative western blot showing total HCN1 expression and surface HCN1 expression isolated by surface biotinylation. Western blotting against the endogenous NKA and GAPDH as membrane and cytosolic protein controls. **E)** Relative amount of total and surface mRuby3-HCN1_Δ3, 14-3-3_ compared to mRuby3-HCN1 normalized to NKA. Error bars represent standard error of mean for electrophysiology data (A, B, C) and standard deviation for western blot data (E). T-test p < 0.05 (*); p < 0.01 (**); p<0.001 (***); p < 0.0001 (****).

To determine if 14-3-3 is required to regulate HCN1 activity, we generated stably integrated HEK293 cell lines expressing either mRuby3-HCN1 or mRuby3-HCN1_Δ3,14-3-3_. In mRuby3-HCN1_Δ3,14-3-3_ the amino acids corresponding to 14-3-3 binding peptides B and C (residues 804–814 and 861–871) were deleted while targeted alanine substitution was performed at the site corresponding to peptide A to prevent phosphorylation while preserving the di-arginine ER retention motif (^647^SRMR**T**Q**S**PPVY to ^647^SRMR**A**Q**A**PPVY) [46]. Whole-cell current recordings from these two cell lines revealed that the voltage of half activation of the mutant HCN1 was similar to the wild-type; mRuby3-HCN1 V_1/2_: -118.6 ± 0.1 mV and mRuby3-HCN1_Δ3, 14-3-3_ V_1/2_: -116.1 ± 0.1 mV (Fig 3A). These V_1/2_ values are much more negative than the value of ~-75 mV that is typically reported for HCN1 expressed in HEK293 cells [i.e. 53, 54]. The likely explanation for this discrepancy is that our recordings used potassium fluoride in the intracellular recording solution. Potassium fluoride has been shown to inhibit PKA to produce a large hyperpolarizing shift in the V_1/2_ of HCN1 in odorant receptor neurons [55, 56]. Given that caveat, we conclude that the HCN1 triple mutant is a functional hyperpolarization-activated channel, and we noted increased current density in cells expressing the mutant HCN1 (maximal current density of mRuby3-HCN1: -34.64 ± 6.37 pA/pF vs mRuby3-HCN1_Δ3, 14-3-3_: -69.10 ± 10.34 pA/pF; p = 0.0047; Fig 3B and 3C). Complimentary surface biotinylation experiments revealed that mRuby3-HCN1_Δ3,14-3-3_ had higher total and surface expression levels compared to mRuby3-HCN1 (total: 2.10 ± 0.30-fold increase compared to mRuby3-HCN1; p < 0.0001 and surface: 2.50 ± 0.10-fold increase compared to mRuby3-HCN1; p = 0.0071; Fig 3D and 3E). These experiments led us to consider that 14-3-3 could be involved in the turnover or trafficking of HCN1. However, if 14-3-3 is involved in regulating the trafficking of HCN1 it is unlikely to be through the mechanism of masking the di-arginine ER export motif, because this mechanism predicts that the mutant channel would have increased retention instead of increased surface expression.

### 14-3-3 interacts with two sites at the distal C-terminus of HCN1

**Fig 4:**
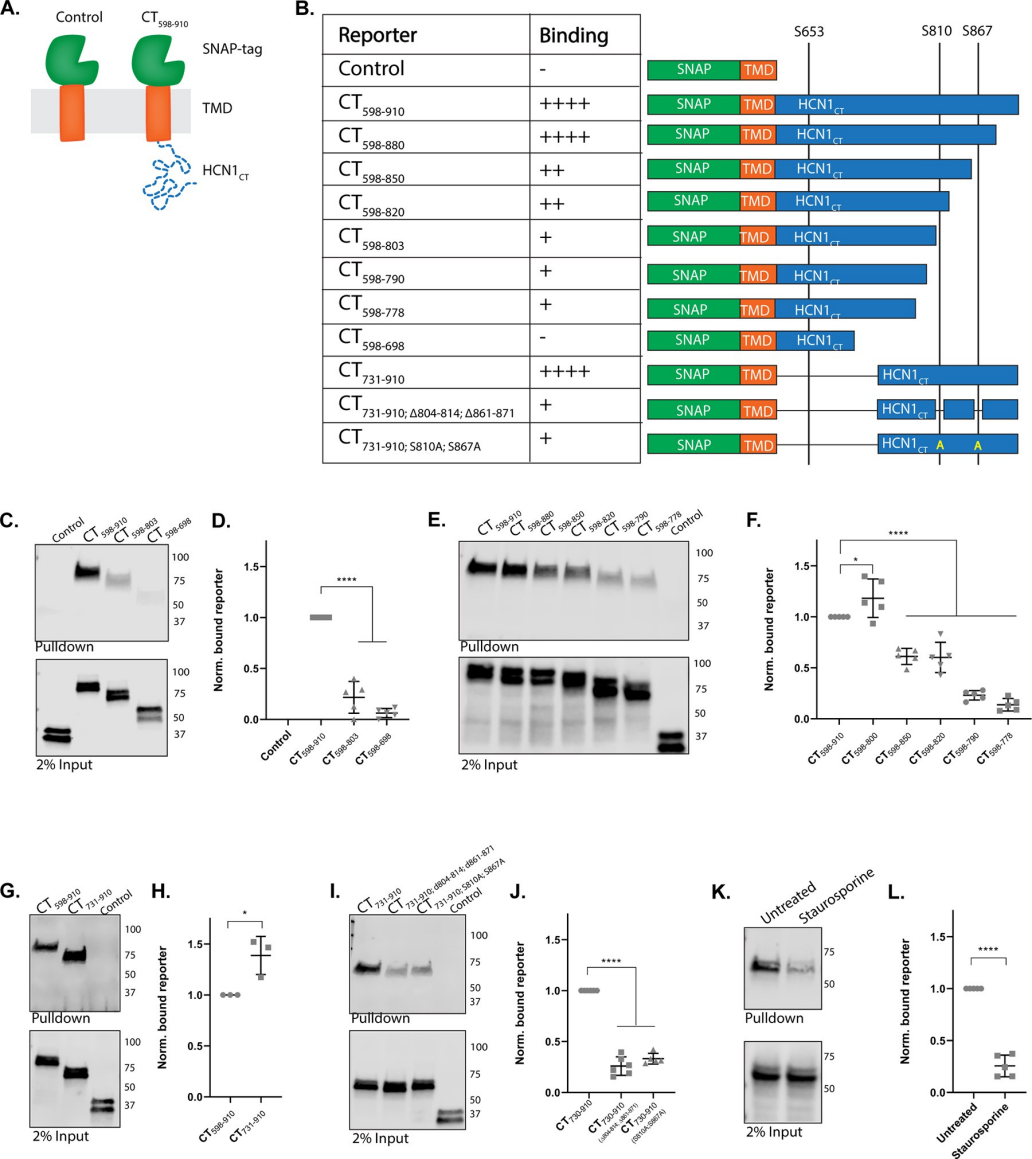
14-3-3 interacts with two sites in the distal C-terminus of HCN1. **A)** Schematic of the reporter system used in this series of experiments. The C-terminus of HCN1 (residues 598–910) was fused to the transmembrane reporter. Reporters were expressed in HEK293 cells and pulled down with recombinant 14-3-3ζ then analyzed by western blotting for the SNAP-tag. **B)** Summary of reporters used. Binding is given as efficiency compared to full-length HCN1 C-terminus with ++++ = 100%, +++ = 75%, ++ = 50%, + = 25%, and— = 0% binding efficiency. **C, E, G, I)** Representative blots and comparisons of normalized results **(D, F, H, J)** for pulldowns of the reporter-HCN_CT_ constructs as indicated**. K)** representative pulldown **L)** comparison of pulldown from cells CT_730-910_ treated with the broad-spectrum kinase inhibitor staurosporine. For each construct, the p-value or adjusted p-value for comparison to CT_598-910_ or CT_731-910_ or untreated control is shown p < 0.05 (*); p < 0.01 (**); p<0.001 (***); p < 0.0001 (****).

**Fig 5:**
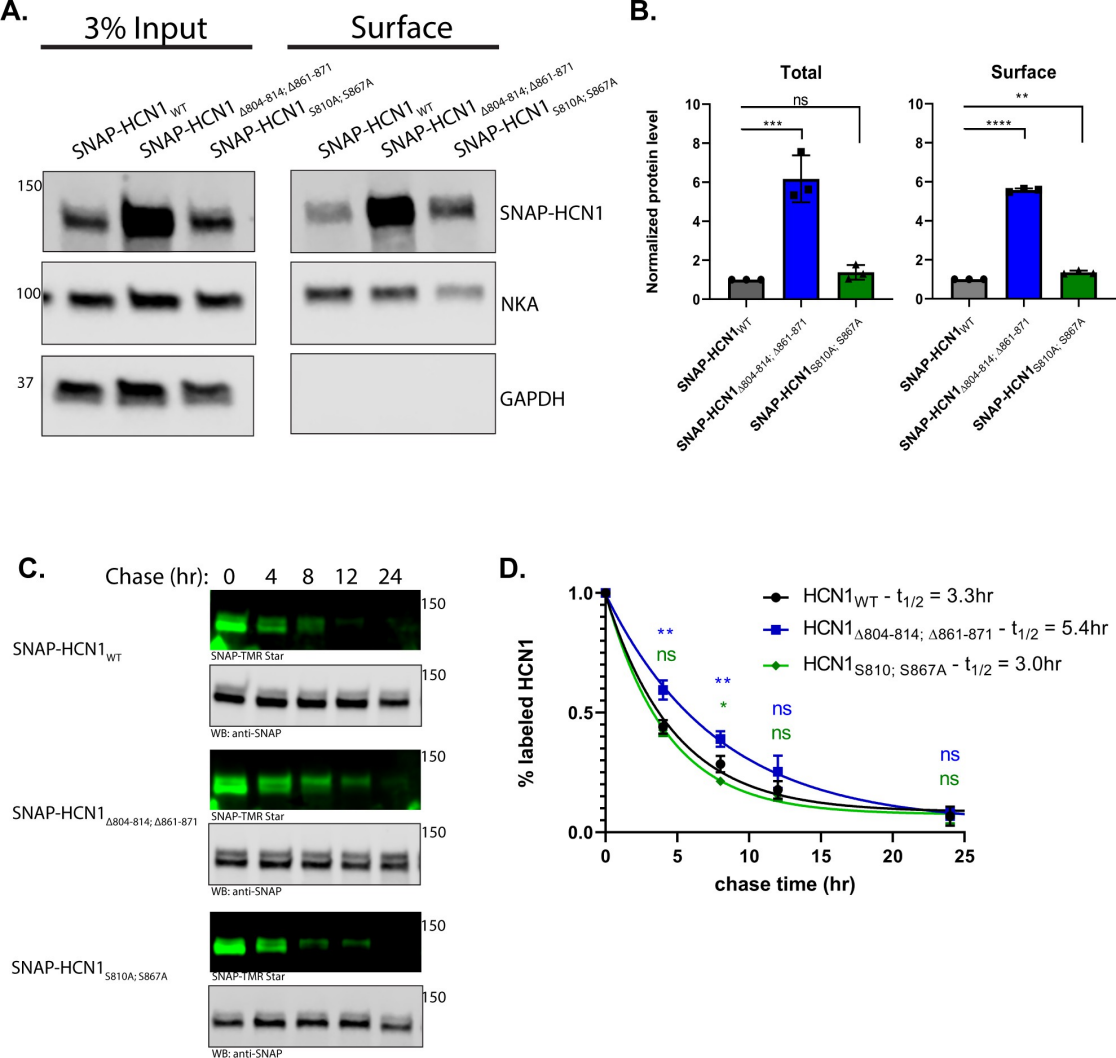
14-3-3 interaction sites regulate HCN1 turnover in a phosphorylation independent manner. **A)** Representative western blot showing total SNAP-HCN1 expression and surface SNAP-HCN1 expression isolated by surface biotinylation with western blotting against the endogenous NKA and GAPDH as membrane and cytosolic protein controls. B) Relative total and surface level of SNAP-HCN1_Δ804–814; Δ861–871_ (blue) and SNAP-HCN1_S810A; S867A_ (green) compared to SNAP-HCN1 (black) normalized to NKA. **C)** Representative fluorescence imaging of SNAP-HCN1 harvested after 0, 4, 8, 12, and 24 hours of chase. SNAP-TMR Star was used to detect labeled SNAP-HCN1 and anti-SNAP antibody for total SNAP-HCN1. **D)** Relative amount of labeled SNAP-HCN1 (black), SNAP-HCN1_Δ804–814; Δ861–871_ (blue), and SNAP-HCN1_S810A; S867A_ (green), normalized to amount at 0 hour chase plotted against chase time to give a decay curve. The adjusted p-value for comparison to SNAP-HCN1 is shown p < 0.05 (*); p < 0.01 (**); p<0.001 (***); p < 0.0001 (****).

To determine if all three 14-3-3 binding sites are used in living cells, and to assay for any additional sites 14-3-3Pred may not have scored highly, we used transiently transfected HEK293 cell lysates as a source of HCN1 protein fragments in pull down assays with immobilized _6His_14-3-3ζ. We first tested if the C-terminus of mouse HCN1 was sufficient for 14-3-3 association. Amino acids 598–910 of HCN1 (CT_598-910_) was fused to a transmembrane domain (from the PDGF-receptor) with an extracellular SNAP-tag as shown in Fig 4A. CT_598-910_, but not the control base reporter, was pulled down by 14-3-3 (Fig 4C and 4D). Serial truncations of the HCN1 fragment demonstrated that loss of residues 803–910 (containing peptides B and C) severely limited the interaction (0.20 ± 0.10-fold decrease compared to CT_598-910_; p < 0.0001). Further deletion that left peptide A intact (CT_598-698_) resulted in complete loss of interaction (0.06 ± 0.04-fold decrease compared to CT_598-910_; p < 0.0001) (Fig 4C and 4D). The complimentary deletion of the peptide A-containing region confirmed it was not necessary for 14-3-3 association (Fig 5G).

Serial deletions of 30 amino acid blocks from the most distal portion of the C-terminal tail of HCN1 generated three findings (Fig 4E and 4F). First, deleting the block containing the TRIP8b-tripeptide binding site did not impede the interaction between 14-3-3 and HCN1 as slightly more of this truncated reporter was pulled down (1.18 ± 0.18-fold increase compared to CT_598-910_; p = 0.0359), supporting our earlier decision to disregard the prediction of a fourth putative 14-3-3 binding site. Second, HCN1 fragments lacking the region containing peptide C (amino acids 850–880) resulted in loss of half the pull down efficiency (0.61 ± 0.07-fold decrease compared to CT_598-910_; p < 0.0001). Third, another drop in pulldown efficiency was observed when the serial truncation also resulted in loss of the region containing peptide B (residues 790–820; 0.23 ± 0.04-fold decrease compared to CT_598-910_; p < 0.0001).

To determine whether the distalmost C-terminus of HCN1 was sufficient to mediate the interaction with 14-3-3, the proximal region of the C-terminus including peptide A was removed leaving only a 180 amino acid long fragment of the distal C-terminus that retains the disordered polyQ stretch as a spatial linker (residues 731–910). This construct was pulled down slightly more efficiently than the construct containing the full-length C-terminus (Fig 4G and 4H; 1.39 ± 0.19-fold increase compared to CT_598-910_; p = 0.0227). Finally, site-specific mutations were used to delete just the 10 amino acid blocks corresponding to peptides B and C from the distal C-terminus (CT_731-910; Δ804–814; Δ861–871_) or to mutate the phosphorylation sites of those peptides with serine to alanine replacements (CT_731-910; S810A; S867A_). Both sets of mutations reduced 14-3-3 interaction significantly (Fig 4I and 4J; 0.26 ± 0.09-fold decrease compared to CT_731-910_ for CT_731-910; Δ804–814; Δ861–871_ and 0.33 ± 0.05-fold CT_731-910_ for CT_731-910S; S810A; S867A_; p < 0.0001 for both). This data indicates that in unstimulated HEK293 cells, 14-3-3 associates with two sites in the disordered tail of HCN1 centered around S810 and S897.

### Kinase activity is required for interaction between 14-3-3 and HCN1

We next tested the requirement for kinase activity. HEK293 cells transfected with the reporter CT_731-910_ were treated with the broad-spectrum kinase inhibitor staurosporine for three hours, then tested for interaction with 14-3-3 using the pull-down assay. Staurosporine reduced the efficiency of CT_731-910_ pulldown similar to the S810A; S867A mutation (Fig 4K and 4L; 0.26 ± 0.10-fold decrease compared to untreated control; p < 0.0001). Treatment with more specific kinase inhibitors had lesser effects and generally any single inhibitor slightly reduced the amount of CT_731-910_ pulled down (S1 Fig). Only treatment with the PKC inhibitor (bisindolylmaleimide I) and the p38MAPK inhibitor (PD 169316) had a statistically significant effect on the amount of reporter pulled down by _6His_14-3-3ζ (PKC inhibition: 0.61 ± 0.11-fold decrease compared to untreated control; p = 0.0468 and p38MAPK inhibition: 0.61 ± 0.19-fold decrease compared to untreated control; p = 0.0468). The need for kinase activity is consistent with the canonical mechanism of 14-3-3 recruitment.

### 14-3-3 interacts with a region that negatively regulates HCN1 expression

Having determined that only two of the initially tested HCN1 phosphopeptides are involved in 14-3-3 association in HEK293 cells we tested the effect of more specific mutations on the expression level of HCN1. Stable cell lines were generated for expression of SNAP-tagged full-length mouse HCN1 and two mutant versions of HCN1 –one with both 14-3-3 interaction sites entirely deleted (SNAP-HCN1_Δ804–814; Δ861–871_) and the other with just the central serine residues replaced with alanine, aka ‘phosphonull’ mutants (SNAP-HCN1_S810A; S867A_). HCN1 expression was measured in surface biotinylation assays. We expected both mutant versions of HCN1 would be expressed at ~2 fold higher levels as we saw previously for HCN1_Δ3, 14-3-3_. However, SNAP-HCN1_Δ804–814; Δ861–871_ expressed at roughly six-times the level of SNAP-HCN1 both in total (6.17 ± 1.20-fold increase compared to SNAP-HCN1; p = 0.0003) and at the surface (5.75 ± 0.08-fold increase compared to SNAP-HCN1; p < 0.0001). To our surprise, SNAP-HCN1_S810A; S867A_ did not exhibit a change in total expression and the change in surface expression was small (1.36 ± 0.09-fold increase compared to SNAP-HCN1; p = 0.0018) (Fig 5A and 5B).

To investigate the dynamics of SNAP-HCN1 expression we used pulse-chase assays exploiting the ability of the SNAP-tag to covalently bind substrate. Cells were incubated with a fluorescent SNAP-substrate for 45 min which was then washed out and replaced with a non-fluorescent SNAP-substrate so that any SNAP-HCN1 synthesized after the pulse could not be fluorescently labeled. Cells were harvested at several time points for the next 24 hours. Following SDS-PAGE and transfer onto PVDF membrane, the amount of labeled SNAP-HCN1 was measured by direct fluorescence detection and the amount of total SNAP-HCN1 was measured by western blotting against the SNAP-tag (Fig 5C). The half-life was calculated by fitting the normalized data to decay curves (Fig 5D). The half-life of SNAP-HCN1 was 3.3 hours. The half-life of SNAP-HCN1_S810A; S867A_ was slightly reduced to 3.0 hours and the relative amount of labeled SNAP-HCN1_S810A; S867A_ was decreased slightly compared to SNAP-HCN1 at 8 hours (0.21 ± 0.02 vs 0.28 ± 0.03; p = 0.0278). However, the half-life of SNAP-HCN1_Δ804–814; Δ861–871_ was significantly increased to 5.4 hours with the relative amount of labeled SNAP-HCN1_Δ804–814; Δ861–871_ being elevated compared to SNAP-HCN1 at 4 hours (0.59 ± 0.04 vs 0.44 ± 0.03; p = 0.0021) and 8 hours (0.39 ± 0.03 vs 0.28 ± 0.03; p = 0.0077). A longer half-life indicates increased stability of the protein and would lead to the increased static expression levels we observed.

## Discussion

The key finding of this study is the identification of an interaction between HCN1 and the signaling adaptor 14-3-3. 14-3-3 can interact with two sites centered on S810 and S867 in the intrinsically disordered cytoplasmic C-terminus of mouse HCN1. These 14-3-3 interaction sites also overlap with an independent HCN1 degradation signal.

HCN1 phosphorylation has been implicated in either modulating channel activity or expression, likely depending on which sites are phosphorylated. Yet, the previously characterized HCN1 regulatory proteins are not known to require HCN1 phosphorylation for binding. In a recent phosphorylation site mapping study, one of the residues (S867) we identified as necessary for 14-3-3 association with HCN1 was shown to be phosphorylated *in vivo*. In that study, peptides containing the second 14-3-3 binding site (S810) were not resolved, leaving it untested whether this site is phosphorylated *in vivo* [57]. While S867 phosphorylation was consistently identified in rat hippocampal CA1 tissue, there was no apparent change in the amount of S867 phosphorylation in a rat chronic epilepsy model [57]. Thus, when HCN1 S867 becomes phosphorylated in various neurons requires further investigation. We propose that along with S810, S867 phosphorylation recruits 14-3-3 to HCN1 channels.

We focused on the C-terminus of HCN1 as it contains the most evolutionarily conserved predicted 14-3-3 bindings sites, but it is possible that 14-3-3 may interact with other regions of HCN1. The cytoplasmic N-terminus of HCN is intrinsically disordered, like the distal C-terminal tail, and contains several serine and threonine residues. This includes T39 which was shown to be phosphorylated [57] and lies within a high scoring predicted 14-3-3 binding site. 14-3-3 proteins function as dimers thus can bring different clients together or stabilize transient multi-protein complexes [58, 59]. The simplest interpretation of our data is that a single 14-3-3 dimer interacts with HCN1 such that one monomer is binding phosphorylated S810 and the other monomer is binding phosphorylated S867 in one HCN1 subunit. Alternatively, 14-3-3 dimers could cross-link multiple subunits within one channel (or multiple channels) which would likely influence the stability of the channel. At this point we can’t completely rule out the possibility that 14-3-3 interacts indirectly with HCN1. The serine-to-alanine phosphonull results support a phosphorylation dependent recruitment of 14-3-3, and 14-3-3 directly interacts with phosphorylated serines. But most of our experiments included cell lysates so 14-3-3 could be part of a complex that has an alternative component that binds to HCN1-S810 and HCN1-S867 to recruit 14-3-3. Different modes of 14-3-3 binding could elicit unique effects so it will be important to be mindful of these issues as future experiments move to testing the physiological effects of 14-3-3 mediated regulation in different neuronal populations.

One caveat of this study is the use of HEK293 cells as our primary model system. While a useful system to express the various constructs used in this study and examine the function of HCN1 mutants, we recognize that these cells do not adequately reflect neurons endogenously expressing HCN1. Thus, while the findings presented here implicate S810 and S867 as necessary for the 14-3-3 interaction, additional residues may be phosphorylated and accessible to 14-3-3 *in vivo*. The choice of model system likely explains the ambiguous results we obtained testing the effect of individual kinase inhibitors. That experiment would be better carried out *in vivo*, under physiologically relevant conditions, ideally where phosphorylation can be induced rather than relying on manipulation of any basal phosphorylation of HCN1. That said, it is intriguing that we obtained the strongest effects in HEK293 cells by inhibiting PKC and p38MAPK which have both been implicated in neuronal regulation of HCN1 [37, 60]. Another issue is that we do not know the ratio of basally phosphorylated to unphosphorylated HCN1 in HEK293 cells so it could well be that the unphosphorylated pool of HCN1 dominates which would make the serine to alanine substitutions effectively silent mutations. Phosphomimetic substitutions are often used to examine the function of site-specific phosphorylation; however, it is well characterized that negatively charged amino acids are insufficient to recruit 14-3-3, precluding our ability to use this strategy [61–64].

**Fig 6:**
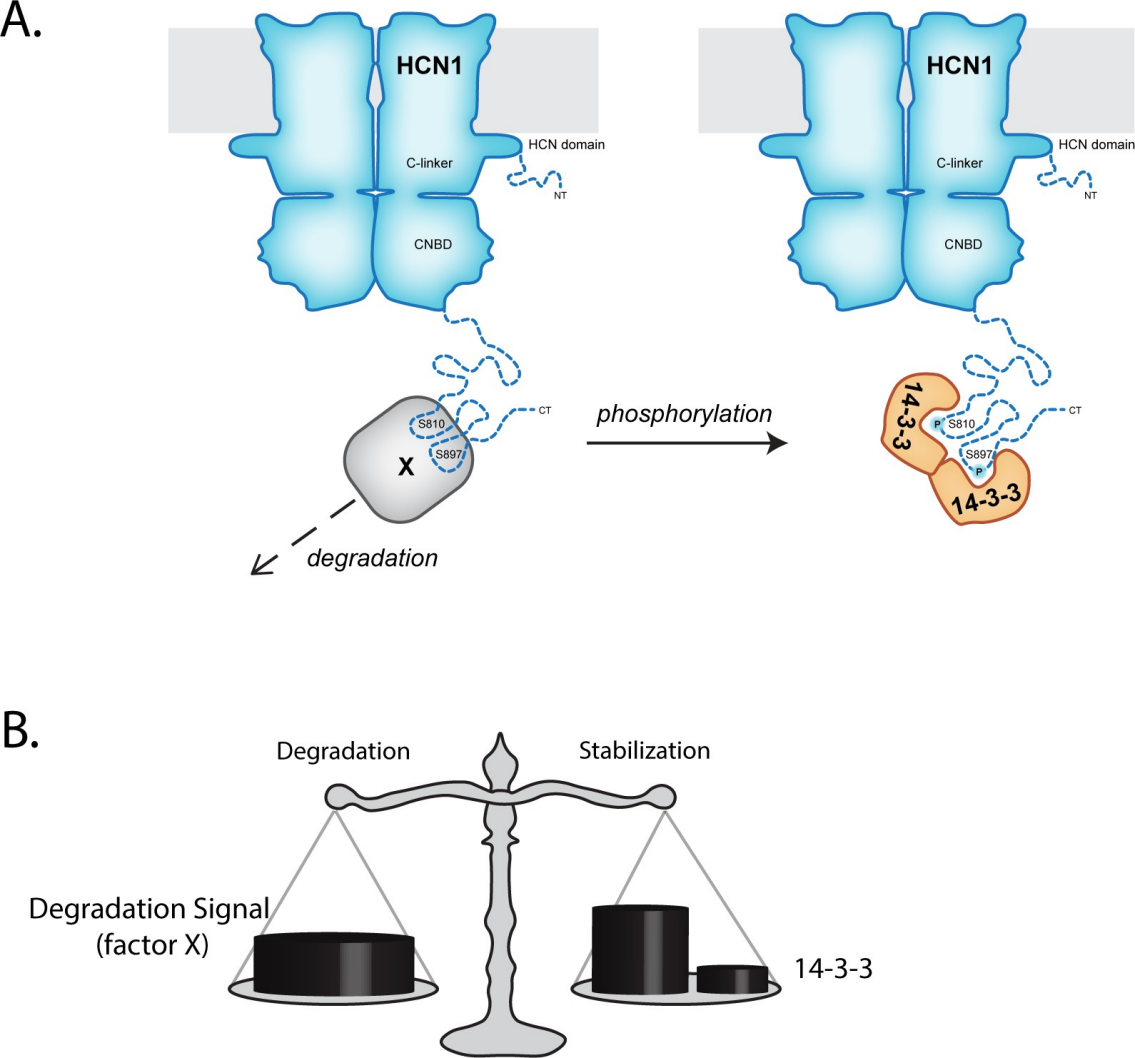
Potential Model for HCN1 degradation versus 14-3-3 interaction with HCN1. **A)** One potential model that could explain the apparent dual nature of the S810 & S867 centered signals in the HCN1 C-terminus–the serines are required for interaction with 14-3-3 and the flanking sequences around those serines participate in HCN1 degradation by an unknown factor (X). HCN1 phosphorylation at S810 and S867 could bias protein-protein interactions at those sites towards 14-3-3 over the Factor X degradation mediator. **B)** HCN1 protein level is established by the balance between destabilizing forces, such as the strong degradation signal, and stabilizing forces. The role of 14-3-3 interaction with HCN1 remains unclear but it could act in combination with other proteins, such as the well-established neuron specific HCN1 binding partner TRIP8b to provide a modularized strong protection signal to balance the effects of the degradation signal.

The major conceptual challenge presented by our data was the observation that 10-amino acid deletions centered on S810 and S867 both prevented 14-3-3 association and decreased the turnover rate of HCN1 leading to increased surface expression and higher current density. While site-specific serine-to-alanine (phosphonull) mutations of S810 and S867 prevented 14-3-3 association but did not significantly affect the static expression levels of HCN1. This indicates that a strong degradation signal is overlapping but distinct from the 14-3-3 interaction with the C-terminus of HCN1. One possibility is that 14-3-3 is a non-essential component of a complex that promotes HCN1 channel degradation. This hypothesis is unsatisfying and suggests that this interaction has no functional role despite S810 and S867 being highly conserved. Instead, we propose that the simplest model involves competition between the degradation signal (working via a yet uncharacterized Factor X) and phosphorylation-mediated recruitment of 14-3-3 (Fig 6). In HEK293 cells, 14-3-3 binding did not overcome the strong degradation signal since loss of only 14-3-3 interaction (via the phosphonull site-specific HCN1 mutants) had a minimal impact on HCN1 turnover (3.0 hr vs 3.3 hr for SNAP-HCN1). But in situations where HCN1 phosphorylation of S810 and S867 is increased, 14-3-3 recruitment could be functioning as part of a modular rheostat for the dynamic control of HCN1 expression.

The region of HCN1 that associates with 14-3-3 is intrinsically disordered, so it is better to think of it as existing in a population of different conformations rather than in just one fixed state. That means binding by multiple proteins may not be competitive when HCN1 channels are considered en masse. Thus, we must consider how the various factors acting on the C-terminus of HCN1 act in coordination, whether they function in competitive or cooperative terms. This is especially important when we consider TRIP8b, an endogenous HCN channel accessory protein absent from HEK293 cells. TRIP8b binds to both the extreme C-terminal Ser-Asn-Leu tripeptide of HCN1 and the cyclic nucleotide binding domain, spanning the intrinsically disordered region containing the 14-3-3 binding sites [29]. Depending on which splice isoform is expressed, this accessory subunit can either promote or inhibit expression of HCN1 such that TRIP8b may act in tandem or opposition with 14-3-3 [30–32]. Another HCN1 interacting partner that must be considered is Nedd4-2 which is a candidate for the unknown signaling factor promoting HCN1 degradation. Nedd4-2 is an E3 ubiquitin ligase known to target multiple ion channels including HCN1 [35, 65]. Nedd4-2 binds the C-terminus of HCN1, though it appears to bind to a more proximal region than the region that interacts with 14-3-3. The lysine ubiquitinated by Nedd4-2 remains unknown and given the flexibility of the disordered C-terminus, may be at a more distal site in closer proximity to the 14-3-3 interaction sites or may depend on the C-terminus adopting a conformation prevented by 14-3-3 recruitment. Testing of these models will be very challenging as it will be most informative if performed using *in vivo* systems that represent normal neuronal homeostasis and responsiveness to neuro-modulatory signaling.

Taken together, this work demonstrates that 14-3-3 interacts with the C-terminus of HCN1 in a phosphorylation dependent manner. Phosphorylation has been implicated as a means to regulate HCN1 channel function and, to our knowledge, this is the first HCN1 interacting partner identified whose binding depends on phosphorylation of the channel. While we are unable to definitively identify the function of this interaction, our work demonstrates that 14-3-3 association occurs at sites involved in regulating HCN1 turnover, identifying this region as an area of interest for further studies into regulation of HCN1 channels. Uncovering all the mechanisms regulating HCN1 is important as this channel is an attractive drug target for amelioration of various neurological and neuropsychiatric conditions.

## Supporting information

S1 Fig 14-3-3 binding requires HCN1 phosphorylation. HEK293 cells expressing reporter-HCN1 CT_731-910_ were treated with kinase inhibitors (detailed in S1 Table) **A)** representative pulldown **B)** comparison of all experimental replicates, dark grey bar is the untreated control, purple bars highlight the treatments that generated a statically significant reduction in pulldown efficiency with adjusted p-value shown.
(TIF)

S1 Table Kinase inhibitors used in Fig 5K and 5L and S1 Fig. (DOCX)

S1 Raw images (TIF)
