# Supplementary figures and images for: Identification of HCN1 as a 14-3-3 client
Source: PLoS One. 2022 Jun 9;17(6):e0268335. doi: 10.1371/journal.pone.0268335 (PMC9182292; doi:10.1371/journal.pone.0268335)

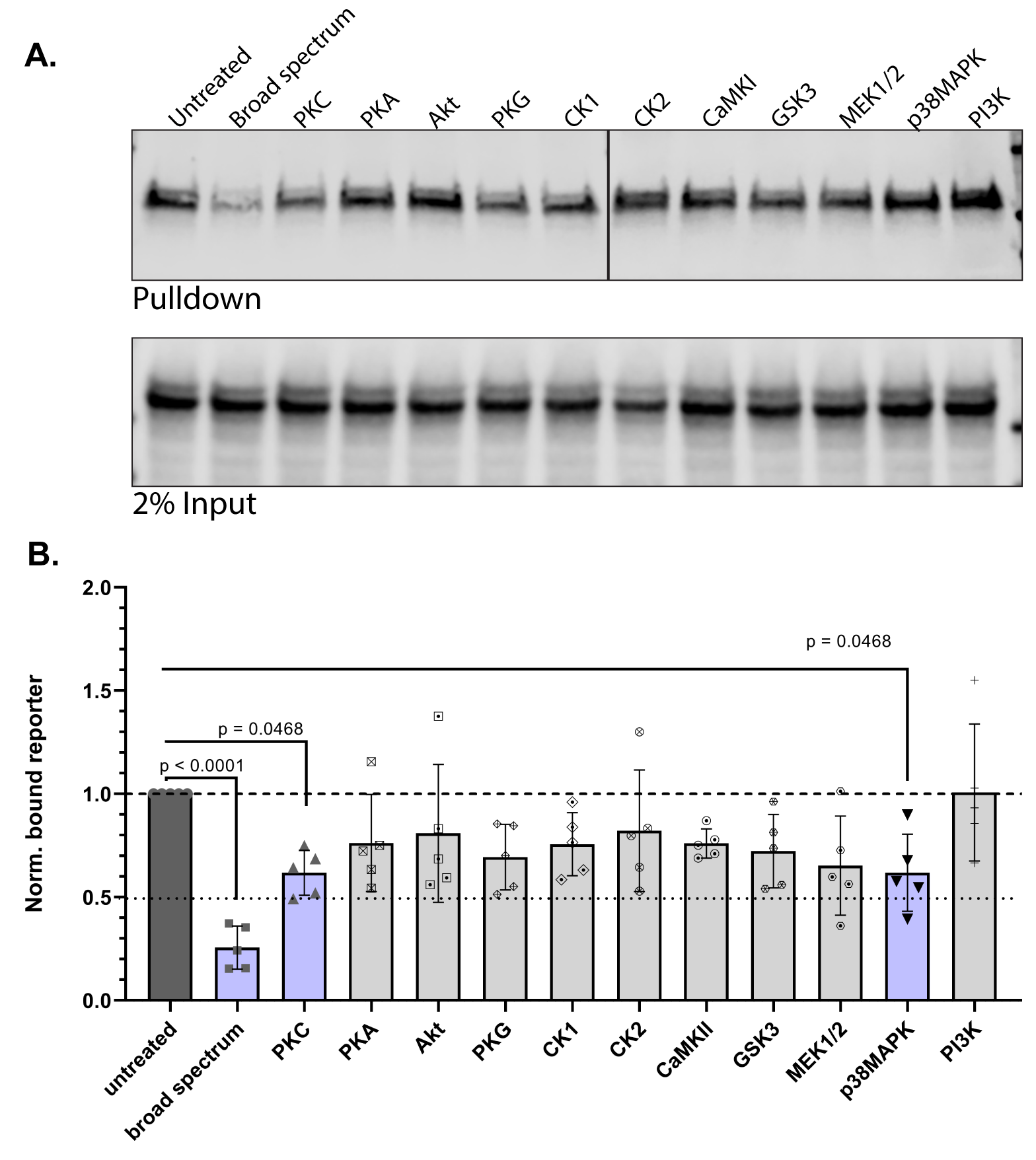

Supplement: S1 Fig — HEK293 cells expressing reporter-HCN1 CT731-910 were treated with kinase inhibitors (detailed in S1 Table) A) representative pulldown B) comparison of all experimental replicates, dark grey bar is the untreated control, purple bars highlight the treatments that generated a statically significant reduction in pulldown efficiency with adjusted p-value shown. (TIF) [file pone.0268335.s001.tif]
