# Supplementary material for: Identification of HCN1 as a 14-3-3 client
Source: PLoS One. 2022 Jun 9;17(6):e0268335. doi: 10.1371/journal.pone.0268335 (PMC9182292; doi:10.1371/journal.pone.0268335)
Supplement: S1 Table — (DOCX) [file pone.0268335.s002.docx]

| **S1 Table: Kinase inhibitors used in Figure 5 (K, L) and S1 Figure** | | | | |
| --- | --- | --- | --- | --- |
| **Compound name** | **Company** | **Cat #** | **Target** | **Concentration (μM)** |
| Staurosporine (Sts) | Millipore – Sigma | 569397 | Broad spectrum | 10 |
| Bisindolylmaleimide I | Millipore – Sigma | 203290 | PKC | 10 |
| H89 | Santa Cruz Biotech. | sc-3537 | PKA | 10 |
| Afuresertib | Cayman Chemicals | 17988 | Akt | 10 |
| H-8 | Cayman Chemicals | 13312 | PKG | 100 |
| D 4476 | Cayman Chemicals | 13305 | CK1 | 20 |
| Emodin | Cayman Chemicals | 13109 | CK2 | 2000 |
| KN-93 | Cayman Chemicals | 13319 | CaMKII | 20 |
| CHIR99021 | Cayman Chemicals | 13122 | GSK3 | 10 |
| U-0126 | Cayman Chemicals | 70970 | MEK1/2 | 10 |
| PD 169316 | Cayman Chemicals | 1006727 | p38MAPK | 10 |
| PI-103 | Cayman Chemicals | 10009209 | PI3K | 10 |
